# Expanding Biolayer Interferometry Applications: Enhanced Accuracy, Precision, and Sensitivity in Residual Biomolecule Detection and Quantitation of Bispecifics and AAV Viral Particles

**DOI:** 10.3390/bios16070390

**Published:** 2026-07-18

**Authors:** Stuart Knowling, Kirsty McBain, David Apiyo

**Affiliations:** 1 Sartorius Bioanalytical Instruments, 47661 Fremont Blvd, Fremont, CA 94538, USA; stuart.knowling@sartorius.com; 2 Sartorius UK Ltd., Grantham Close, York Way, Royston SG8 5WY, UK; kirsty.mcbain@sartorius.com

**Keywords:** Biolayer Interferometry, Octet^®^ BLI, biosensors, cytokines, sensitivity, kinetics, bispecifics, AAV, capsids

## Abstract

Biolayer Interferometry (BLI) has traditionally been used for characterization of protein–protein interactions (PPI) with proteins, such as antibodies and their antigens, through kinetic and quantitation assays. Limitations, for example in sensitivity and the availability of established assay formats, have restricted its adoption across other analytical applications. This article highlights three case studies which demonstrate the expansion of BLI into novel applications, spanning the areas of protein detection and viral vector characterization. The first case study details the use of a multi-step signal amplification assay to enable the detection of low abundant molecules, such as cytokines, at lower concentrations than can be detected using the standard one-step binding approach. Cytokines are sandwiched between biotinylated and HRP-conjugated antibodies, then dipped into 3-amino-9-ethylcarbazole (AEC) reagent, resulting in an enhancement of the cytokine detection sensitivity. The second case study uses BLI for the quantitation of mixed populations of a bispecific antibody (bsAb). Bridging and dual binding assay formats are evaluated for their assessment of bsAb antigen binding kinetics and ability to determine the ratio of correctly assembled bsAb within a sample. In the third case study, BLI detection principles are used to estimate the percentage full capsids in a mixed population of AAV particles. Collectively, these case studies demonstrate the versatility of Octet^®^ BLI and highlight its potential to support an increasing range of analytical workflows beyond its traditional applications.

## 1. Introduction

Biochemical analysis has traditionally been performed using techniques such as enzyme linked immunosorbent assays (ELISA), Western blotting and high-performance liquid chromatography (HPLC) [1,2]. The trend, however, has rapidly shifted towards the adaptation of label-free analytical techniques that can convert biomolecular interactions into response signals that can be measured in real time, allowing researchers to detect anomalies in detection that can in turn be rapidly optimized [3]. Label-free systems have become useful tools in kinetics and affinity characterization, concentration determination and biomolecular interactions screening. In kinetics and affinity characterization assays, label-free technologies provide real-time information about the association (*k*_a_, *k*_on_) and dissociation (*k*_d_, *k*_off_) rate constants, which are key determinants in determining the global affinity (*K*_D_) of an interaction [4]. Importantly, end-point analysis techniques such as ELISA cannot determine the association and dissociation rate constants, so label-free technologies offer a major advantage when elucidating biomolecule interactions [4]. Commonly used label-free technologies such as Isothermal Titration Calorimetry (ITC), Surface Plasmon Resonance (SPR) and Biolayer Interferometry (BLI), each offer unique advantages that make them appropriate for specific applications [5]. BLI, through Octet^®^ platforms, has become a popular technique for antibody titer determination and is now an essential technology, especially in high-throughput antibody production laboratories [4]. In addition to titer, Octet^®^ BLI is commonly used for kinetics and affinity characterization of biological molecules, in both upstream and downstream drug discovery, reagent development laboratories and regulatory approved medicines [6,7]. In this article, we detail case studies where the use of BLI has been extended to non-traditional applications. These examples demonstrate that with the appropriate assay design and optimization, there is an ever-increasing potential for widespread application of BLI across previously inaccessible assay formats.

## 2. Principles of Biolayer Interferometry (BLI)

**Figure 1:**
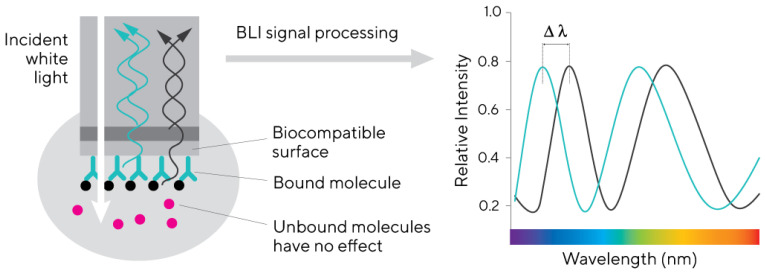
Relative intensity of the light reflection pattern from the two surfaces on the biosensor. BLI systems measure the difference in reflected light’s wavelength (Δλ) between the two surfaces.

Biolayer Interferometry (BLI) is an optical analytical technique that utilizes white light shown over a reflective biosensor surface to monitor biomolecular interactions. Incident white light from a detector located above a glass fibre biosensor generates two reflection patterns from two surfaces on the biosensor, a reference surface and a chemistry surface (ligand–analyte complexation). The two reflection intensities are correlated with the refractive index of the layer and a fixed phase shift reflection to obtain a reflectance signal as a function of wavelength [8]. The binding between an immobilized ligand and an analyte in solution produces an increase in optical thickness on the tip of the biosensor that can be measured as a wavelength shift from the reference surface (Figure 1). The difference in λmax (nm) between the interference pattern from the reference surface and that of the chemistry surface as a function of time is recorded and indicates the presence or absence of binding. In this regard, it is uniquely different from Surface Plasmon Resonance (SPR) in that BLI does not rely on refractive index changes in the immediate vicinity of the biosensor surface caused by binding events between two molecules [8]. Rather the BLI binding response signal is determined by optical thickness changes in the biocompatible surface (Figure 1) that result from the complexation of two molecules, and which may be directly correlated to the size of the molecule [8]. Only molecules binding to or dissociating from the biosensor surface can shift the interference pattern and generate a response profile. Unbound molecules, changes in the refractive index of the surrounding medium, or changes in flow rate do not affect the interference pattern. This is a unique characteristic of BLI [8]. Due to this, Octet^®^ BLI has found wide usage in antibody kinetics and quantitative characterization applications.

## 3. Octet^®^ BLI Signal Amplification for Detection of Lowly Abundance Analytes

**Figure 2:**
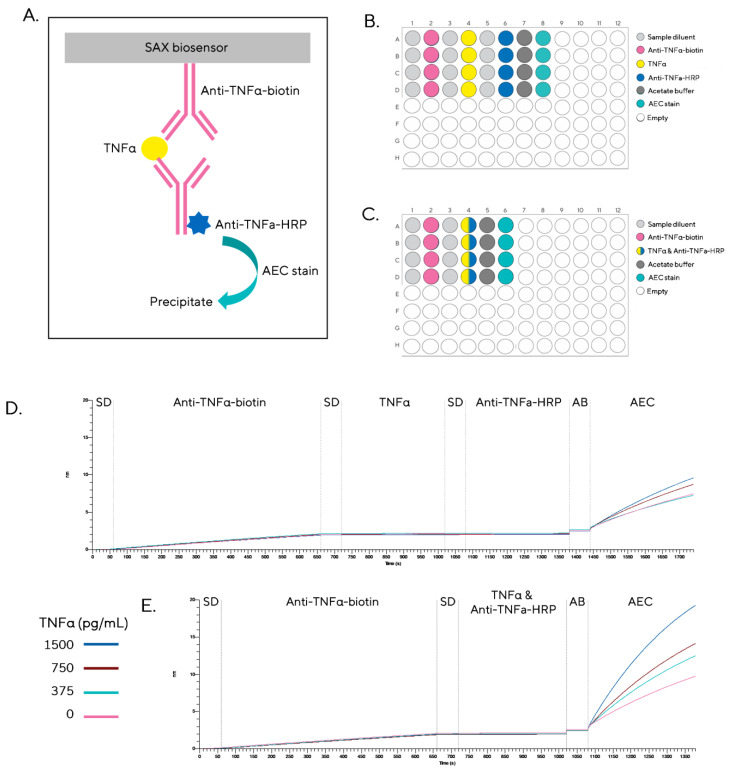
Combining sample and HRP-conjugated antibody steps increases signal window and improves assay sensitivity. (**A**) Schematic of multi-step method used for amplification of signals for detection of low concentrations of TNFα using BLI. (**B**) Plate map for multi-step detection of TNFα (fully separated workflow). (**C**) Plate map for multi-step TNFα detection with sample (i.e., TNFα) and anti-TNFα-HRP steps pre-combined in plate before running on instrument. Traces show detection of a range of concentrations of TNFα (375 to 1500 pg/mL) using (**D**) fully separated and (**E**) combined sample and HRP antibody steps. SD = Octet^®^ Sample Diluent, AB = Sodium Acetate Buffer, pH 5.5.

When quantifying high sample concentrations or when working with large molecules, such as antibodies, BLI technology employs a single biosensor dip step into the sample (contained in a micro-titer plate), which is often sufficient to generate a measurable signal for analyte quantitation. However, when the concentration of the analyte of interest is low, as is common with samples such as biomarkers or residual contaminants, the single-step approach often does not often generate an appreciable binding signal above the signal-to-noise ratio to result in a quantifiable result. A multi-step assay with a signal amplification step is typically needed to resolve this problem and is routinely used in host cell protein (HCP) detection assays. The multi-step assay on BLI is designed as a sandwich format assay, involving the capture of a biotinylated monoclonal antibody, specific to the analyte of interest, to streptavidin-coated biosensors, followed by a dip of the biosensors into the sample of interest (Figure 2A). A matched pair monoclonal HRP-conjugated detection antibody (which binds to a different epitope on the analyte than the capture antibody) is bound next. Biosensors are then dipped into a Horseradish Peroxidase (HRP)- reacting substrate such as metal-enhancing 3,3′-diaminobenzidine (DAB) or 3-amino-9-ethylcarbazole (AEC). The presence of the analyte in the sample results in the oxidation of the AEC substrate through the bound HRP conjugate leads to the formation of an insoluble precipitate which is proportional to the concentration of analyte present. The presence of the insoluble precipitate results in signal amplification and subsequent enhancement of low-concentration analyte detection. While this approach has been extensively used in HCP detection on BLI, it has not found widespread use with other low abundant analytes. In this article, we extend these signal amplification principles to the detection of cytokines, including TNFα and IL-6.

Previous work showed that combining the sample and HRP-conjugated antibody steps in the sample plate prior to assessment using the Octet^®^ can lead to enhanced detection sensitivity compared to running the two steps sequentially on the instrument. Plate maps in Figure 2B,C demonstrate these two assay configurations. Figure 2D,E show the detection of a range of TNFα concentrations (375 to 1500 pg/mL) using the fully separated assay and the combined sample–HRP antibody assay, respectively. There was both a higher maximal signal and window between the top concentration of TNFα tested and the 0 pg/mL reference using the combined-step workflow. There was also greater separation of the lower TNFα concentrations, indicating increased detection sensitivity.

**Figure 3:**
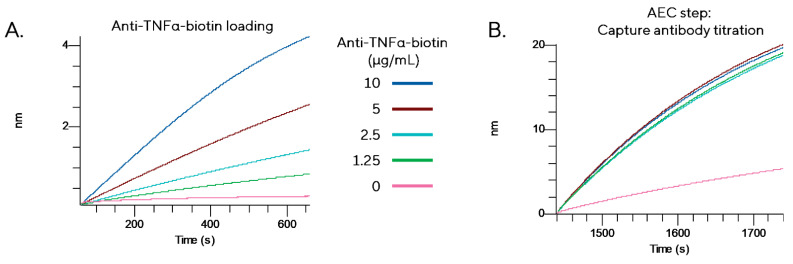
Antibody titration to determine biosensor optimal load concentration. (**A**) Loading of a range of concentrations of biotinylated anti-TNFα (1.25 to 10 µg/mL) (**B**) Signal for AEC amplification step following detection of a single, high concentration of TNFα (10,000 pg/mL) and HRP-conjugated antibody binding to sensors loaded as in panel (**A**).

To determine the optimal concentration of capture antibody, biotinylated anti-TNFα (SinoBiological #10602-MM01, Beijing, China) was loaded onto streptavidin biosensors at varying concentrations. Figure 3A shows the binding response for a range of concentrations of biotinylated anti-TNFα (1.25 to 10 µg/mL, 5:1 molar coupling ratio (MCR)) binding to Octet^®^ SAX biosensors. There is a clear increase in biosensor binding (loading) with increasing antibody concentration. Antibody-loaded biosensors were used in the multi-step assay for TNFα detection, with the final AEC step shown in Figure 3B. All four loaded biosensors showed comparable responses for detection of 10,000 pg/mL TNFα, approximately 4 times larger than the signal for the unloaded reference sensor (i.e., the background signal in the absence of capture ligand). The 5 µg/mL load concentration was used due to the marginally higher binding response to TNFα. The effect of titrating the HRP-conjugated antibody (SinoBiological, #10602-MM08) was also tested by comparing the signal window in the presence and absence of TNFα and 5 µg/mL was deemed optimal.

To test TNFα detection at the determined 5 µg/mL capture antibody concentration, two different HRP substrates, AEC and metal-enhanced DAB, were evaluated for amplification in the presence of HRP antibody conjugate.

**Figure 4:**
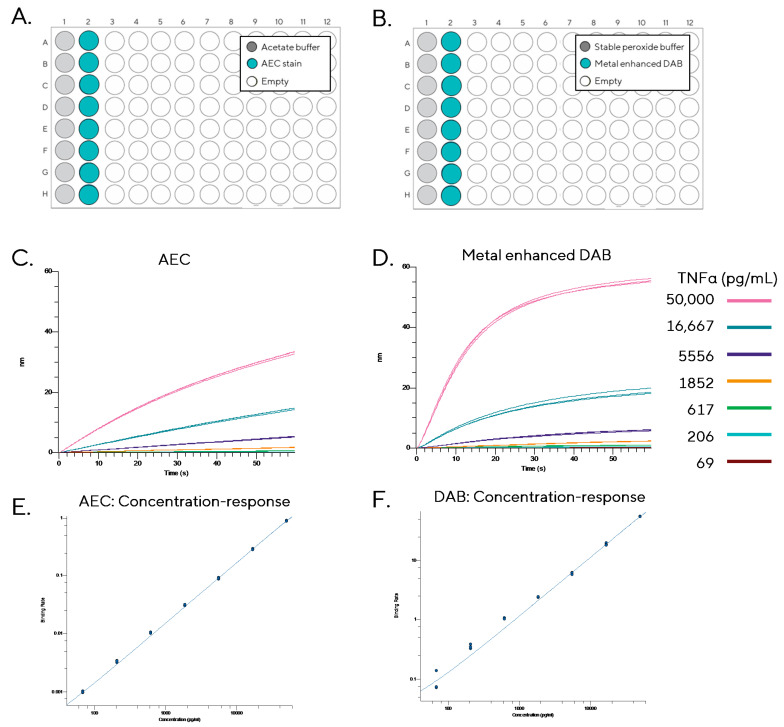
Sensitivity of TNFα detection was higher using AEC compared to metal-enhanced DAB for enzymatic amplification. Initial loading and combined sample and HRP antibody steps were carried out offline using the Octet^®^ AS, an offline biosensor immobilization station. Three replicate columns of biosensors were incubated with a range of concentrations of TNFα (69 to 50,000 pg/mL). A 0 pg/mL reference sensor was included in each column. Plate maps show online amplification steps for (**A**) AEC stain and (**B**) metal-enhanced DAB (buffer and reagent wells were re-used for each column of sensors). Overlay of reference subtracted signals for (**C**) AEC and (**D**) metal-enhanced DAB (*n* = 3). Concentration–response curves with a 5PL unweighted fit for (**E**) AEC (generated using initial slope binding rate equation) and (**F**) metal-enhanced DAB (generated using R-equilibrium binding rate equation). Tables show CVs, % recovery (calculated concentration as a percentage of known concentration), residuals and R^2^ values for AEC and DAB.

The binding rate equation which resulted in an optimal fit was selected for each reagent, with data analyzed using initial binding slope for AEC and binding R-equilibrium for metal-enhanced DAB. Binding responses shown in Figure 4C,D show three replicates at each concentration of TNFα with the 0 pg/mL reference subtracted. These data indicate that the maximum signal for the top concentrations of TNFα is higher with metal-enhanced DAB than with AEC. However, further inspection of the curve fits in the concentration–response curves in Figure 4E,F shows a better quality fit, particularly for the lower concentrations of TNFα (69 to 1852 pg/mL), with the AEC reagent.

Improved sensitivity detection of low TNFα concentrations with the AEC reagent was reinforced with the metrics in Table 1 and Table 2, with CVs < 5% at each concentration, showing highly reproducible data across the concentration range. At lower concentrations of DAB, CVs were higher, particularly at the 69 pg/mL TNFα concentration, which gave a CV of 49% (<10% is considered a good threshold for reproducible quantitation in BLI). The percentage recovery metric showed the calculated concentration as a percentage of the known concentration, to give a measure of how predictive the standard curve is. Generally, above 10% or below 100% is considered acceptable for BLI quantitation assays. Most values for AEC were within this range with only the 206 and 617 pg/mL values reaching slightly higher at 111% and 112%, respectively. In contrast, for DAB, at the same concentrations, the percentage recoveries were much higher at 147% and 143%, respectively. Both the residuals and R^2^ values at low concentrations showed better fits for the AEC amplified data and provided further evidence for improved sensitivity detection of TNFα using this reagent. AEC also has an added benefit of reduced toxicity, which can improve safety and ease of use when compared to the highly toxic metal-enhanced DAB.

To determine the applicability of this amplification assay set-up for detection of other low abundant analytes, experiments were conducted using an anti-IL6 matched antibody pair from SinoBiological (capture antibody #10602-MM01 and detection antibody #10602-MM08). Most steps were transferred directly from the TNFα assay; however, the antibody titration experiments were repeated to ensure optimal concentrations were used for this pair. In this instance, much lower concentrations of 0.625 µg/mL and 2.5 µg/mL (compared to 5 µg/mL for the TNFα assay) were found to be optimal for the biotinylated antibody and HRP-conjugated antibody, respectively. The time for the AEC step was also extended to 300 s for the IL-6 assay, as the traces still increased linearly by the end of the 60 s timeframe used for TNFα experiments.

**Figure 5:**
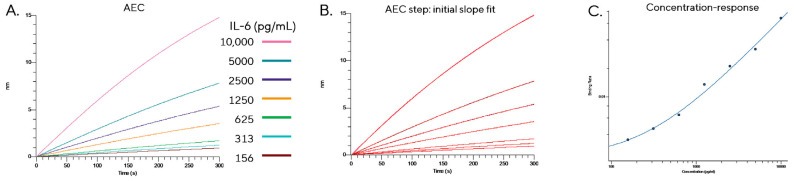
Amplification workflow can be applied to other molecules, such as IL-6. (**A**) Reference subtracted signals for AEC amplification step showing detection of a range of concentrations of IL-6 (156 to 10,000 pg/mL). (**B**) Initial slope binding rate equation applied to the AEC step. (**C**) Concentration–response curve calculated by applying initial slope binding rate equation to A and fitting with 5PL unweighted curve.

Figure 5A shows the AEC amplification step for detection of a range of concentrations of IL-6 (156 to 10,000 pg/mL) using optimized conditions. An initial slope binding rate equation (Figure 4B) was applied and used to generate the concentration response–curve in Figure 4C (5PL unweighted). R^2^ values for this curve were >0.999.

These two case studies show that signal amplification using an enzyme reaction step can be used to extend BLI low abundance analyte detection to cytokines such as TNFα and IL-6. Detection of cytokines is critical in in vitro research for characterization of immune cell function, therapeutic evaluation and predicting inflammatory responses [9,10].

## 4. Bispecific Antibody Detection and Quantitation Using Octet^®^ BLI

Bispecific molecules are engineered to bind two distinct targets simultaneously and offer a strategic advantage in therapeutic applications, particularly in complex diseases such as cancer, autoimmune disorders, and infectious diseases. The dual-targeting capability of bispecific molecules is highly advantageous as it enhances specificity whilst reducing off-target effects, making them highly desirable in modern drug development.

A fundamental issue in the generation of bispecific IgG antibodies is the random association of heavy and light chains [11]. Heterodimerization of the heavy chain was resolved using knob-into-hole (KiH) but correct association of the light chains remained a protein engineering problem. A major advancement in the ability to enforce correct light chain association in IgG antibodies was made by a multi-group team from Roche in 2011 who first described the use of CrossMab technology [12]. Despite these advancements, non-functional side products such as monospecific antibodies or Fab from the parental antibodies can still be an issue when engineering bispecific (and multispecific) antibodies.

Here, we demonstrate how Octet^®^ BLI can be used to assess multiple parameters of bispecific binding, including whether antigen binding is independent or sequential, and methods to assess the ratio of correctly produced bispecific antibodies. Two approaches, bridging and dual binding assay methods, are discussed.

All assays were performed in 1× HBS-EBT, which was prepared from 1× HBS-EP+ supplemented with 1 mg/mL BSA. Octet^®^ SAX Biosensors were used for assessment of binding in the bridging format and Octet^®^ AHC2 Biosensors were used for all dual binding assays assessment of HER2 and HER3 binding to zenocutuzumab. All biosensors were hydrated for at least 30 min at room temperature prior to use in 1× HBS-EBT in a 384-well, black, tilted bottom microplate. All assays were performed at 25 °C.

### 4.1. Analyte Preparation

For the assessment of the bridging assay format using Octet^®^ BLI, Zenocutuzumab (MedChemExpress catalogue no. HY-P99507, Monmouth Junction, NJ, USA), HER2 (His & Avitag™) and HER3 (His Tag) were used. HER2 (His & Avitag™) and HER3 (His Tag) were prepared to a final concentration of 2.5 µg/mL and 20 µg/mL (200 nM) in 1× HBS-EBT, respectively. Zenocutuzumab was prepared to a final concentration of 10 µg/mL in 1× HBS-EBT. A reference sample of 1× HBS-EBT was used in all assessments to correct for baseline drift.

The bridging assay format assessment was also performed on Octet^®^ SPR using zenocutuzumab, HER2 (His & Avitag™) and HER3 (His Tag). HER2 (His & Avitag™) and HER3 (His Tag) were prepared to a final concentration of 2.5 µg/mL and 20 µg/mL (200 nM) in 1× HBS-EBT, respectively. Zenocutuzumab was prepared to a final concentration of 10 µg/mL in 1× HBS-EBT. The sensor chip surface was activated with a 1:1 mixture of 0.4 M 1-ethyl-3-(3-dimethylaminopropyl)-carbodiimide (EDC) and 0.1 M N-hydroxysuccinimide (NHS). A total of 50 µg/mL streptavidin (diluted in 10 mM acetate pH 4.5) was injected for 6 min with a flow rate of 10 µL/min. After blocking with 1 M ethanolamine–HCl pH 8.0, approximately 1500 RU of streptavidin was immobilized. Following immobilization of HER2 (His & Avitag™), zenocutuzumab was captured using a flow rate of 5 µL/min for 300 s and HER3 was then injected over the surface for 240 s at a flow rate of 30 µL/min prior to a final dissociation period of 300 s. Note that the assessment on SPR was performed for comparison to BLI as this assay format has previously been successfully deployed on the SPR platform [13].

For the assessment of the dual binding assay format Zenocutuzumab, HER2 (His Tag) and HER3 (His Tag) were used. HER2 (His Tag) and HER3 (His Tag) were prepared to a final concentration of 5 µg/mL (50 nM) and 20 µg/mL (200 nM) in 1× HBS-EBT, respectively. Zenocutuzumab and Her2-hOKT3 BsAb-L were prepared to a final concentration of 10 µg/mL in 1× HBS-EBT. A reference sample of 1× HBS-EBT was used in all assessments to correct for baseline drift.

### 4.2. Bridging Assay Format

**Figure 6:**
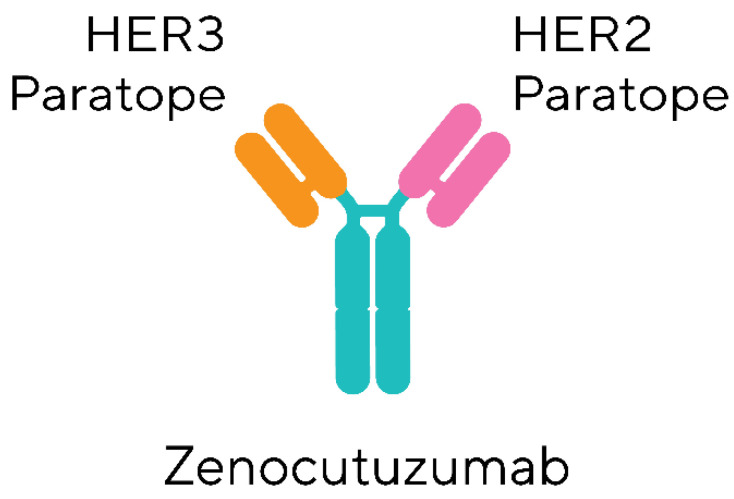
Zenocutuzumab is a humanized IgG1 bispecific antibody that contains paratopes for HER2 and HER3 and induces cell death through antibody-dependent cellular cytotoxicity (ADCC) [15].

In order to assess bispecific assays on Octet^®^ BLI, a biosimilar of the recently approved bispecific Bizengri^®^ (zenocutuzumab) was used (MedChemExpress). Zenocutuzumab is a humanized IgG1 bispecific antibody that contains paratopes for HER2 and HER3 (Figure 6) and is approved specifically for patients with advanced, unresectable, or metastatic non-small cell lung cancer (NSCLC) [14,15].

**Figure 7:**
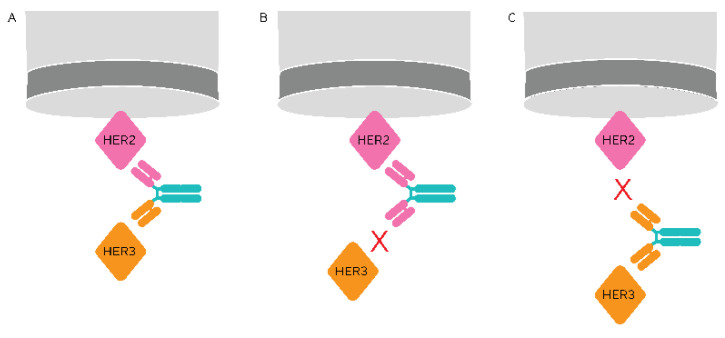
In the bridging assay format only the correctly produced bispecific antibody that contains both paratopes for its target antigens can form a bridge between antigens (**A**). Where the bispecific antibody does not contain either paratope, lack of binding to either antigen is observed (**B**,**C**).

Initial assessment of zenocutuzumab focused on using a bridging assay format (Figure 7), which was originally proposed by Gassner et al. [16]. The bridging assay format involves three stages: antigen 1 immobilization, bispecific antibody capture and antigen 2 binding. As only the bispecific antibodies that contain both paratopes are able to bind both antigens, the assay reflects the active concentration of the bispecific antibody created (Figure 7).

**Figure 8:**
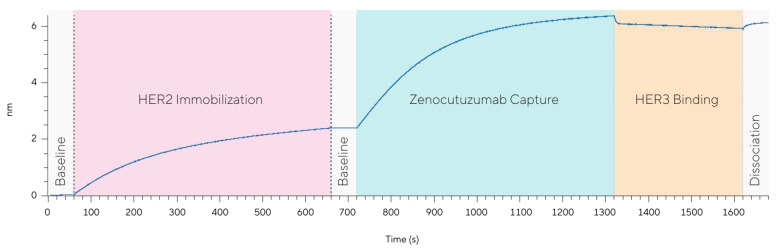
Initial assessment of zenocutuzumab using the bridging assay format on Octet^®^ BLI revealed no binding to the HER3 analyte in solution.

Initial assessment of the bridging assay format with HER2 (His & Avitag™) as the immobilized ligand and HER3 (His Tag) as the analyte in solution were unsuccessful (Figure 8). Despite adequate response changes (~2 nm) for the initial HER2 (His & Avitag™) immobilization on Octet^®^ BLI SAX biosensors and subsequent zenocutuzumab capture to the immobilized HER2 (His & Avitag™), the analyte HER3 (His Tag) was unable to bind to the secondary arm on zenocutuzumab. This effect was at first considered to be caused by a low affinity for HER3.

**Figure 9:**
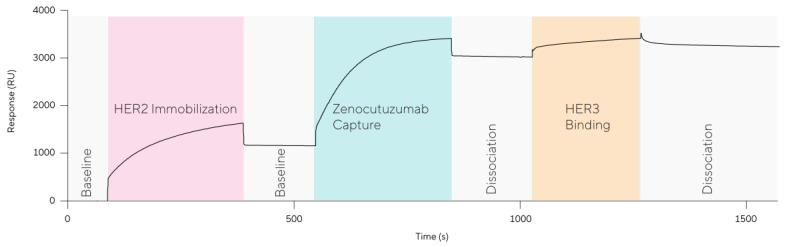
Assessment of zenocutuzumab using the bridging assay format on Octet^®^ SPR revealed zenocutuzumab was capable of binding HER3 analyte in solution.

In order to further investigate the lack of HER3 binding, the same reagents were assessed using surface plasmon resonance. The bridging assay format was performed on an Octet^®^ SF3 using an Octet^®^ SPR CDL Sensor Chip, which comprises ~50 nm carboxymethyl dextran. Following immobilization of streptavidin to the sensor chip surface, HER2 (His & Avitag™) was immobilized and zenocutuzumab captured. HER3 (His Tag) was then injected, and as shown in Figure 9 the expected binding of HER3 to the HER2–zenocutuzumab complex.

The bridging assay format shown by Gassner et al. [16] was initially performed using carboxymethyl dextran SPR sensor chips with a chain length of ~100 nm; therefore, surface structure and chemistry possibly play a role in the lack of HER3 binding. SPR sensor chips contain motile carboxymethyl dextran chains which allow a large amount of conformational freedom of molecular complexes whereas BLI sensors require a more 2D molecular surface close to the biosensor surface in order to observe binding. Therefore, the failure of the bridging assay format using BLI is likely due to steric hinderance of the HER2–zenocutuzumab complex preventing exposure of the HER3 paratope on zenocutuzumab binding to the HER3 analyte.

### 4.3. Dual Binding Assay Format

**Figure 10:**
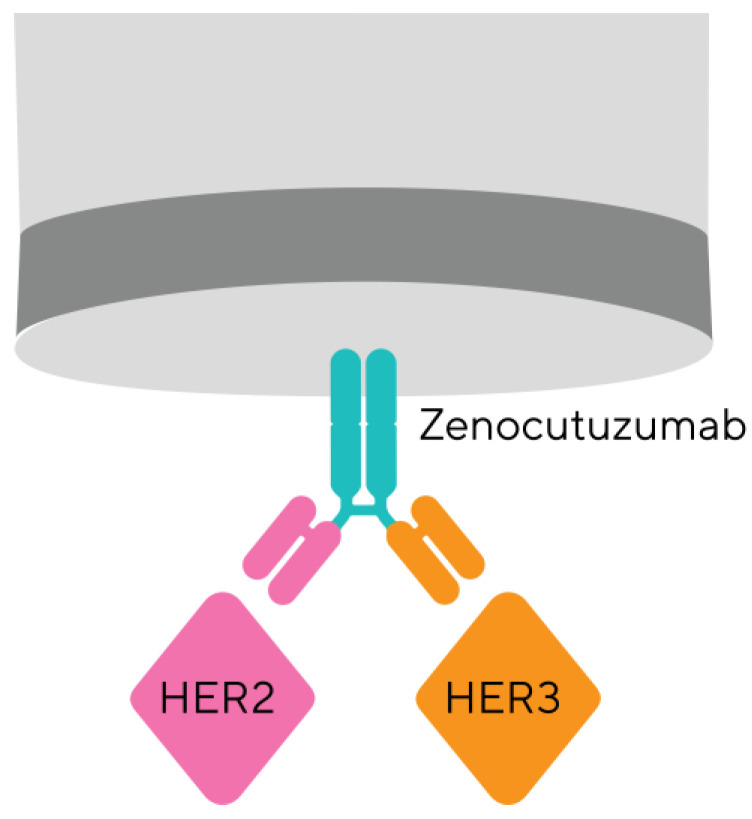
Dual binding assay format. As with the bridging assay format, only bispecifics that contain both paratopes for its target antigens can successfully bind both antigens. Lack of binding to either antigen in solution will show no increase in binding response above baseline.

Although the bridging assay format offers a simple and highly informative assay potential, pitfalls were highlighted by Meschendoerfer et al. and an alternative assay format was suggested [17]. As shown in Figure 10, the dual binding assay format allows individual and simultaneous assessment of each antigen binding to a bispecific assay during a single assay. Advantages of the dual binding assay format include simplified regeneration conditions due to the use of standard capture biosensors (here Octet^®^ BLI AHC2), which can increase assay throughput where required. As highlighted by Meschendoerfer et al. [18], the dual binding assay format allows the contribution of each individual binding event to the overall binding response to be assessed during the assay.

Initial assessment of HER2 and HER3 binding to zenocutuzumab was performed in order to determine whether the binding of each antigen occurs independently or whether binding of HER2 causes an increase/decrease in HER3 binding or vice versa.

**Figure 11:**
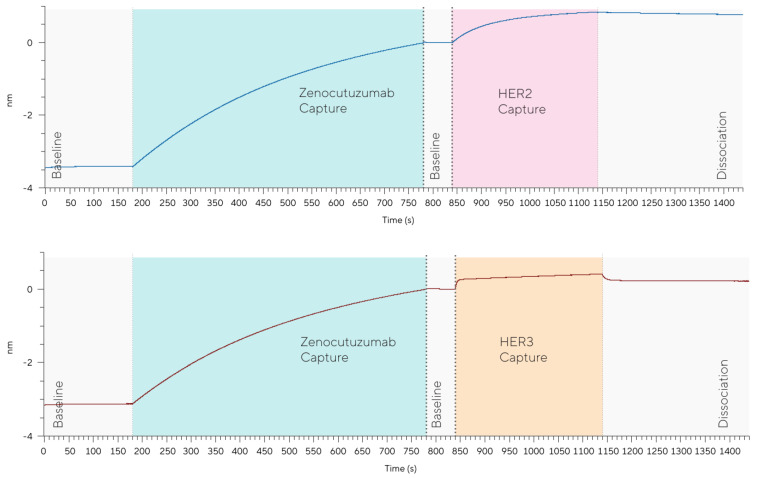
Single binding of HER2 (**top**) and HER3 (**bottom**) shows zenocutuzumab is capable of binding both antigens using the dual binding assay format. Sensorgrams normalized to zenocutuzumab capture response.

As shown in Figure 11 and Table 3, when using the alternative dual binding assay format, zenocutuzumab clearly binds both HER2 (Figure 11 top) and HER3 (Figure 11 bottom).

Although zenocutuzumab can bind both HER2 and HER3 when either is presented independently, it is important to determine whether these binding events occur independently of one another or whether binding of one antigen affects the binding response level of the second antigen.

As shown in Table 3, the individual binding response of HER2 and HER3 to zenocutuzumab was 0.8884 and 0.4398, respectively. If antigen binding to zenocutuzumab is independent, then the final binding response of ~1.3 nm would be expected when sequential binding is assessed.

**Figure 12:**
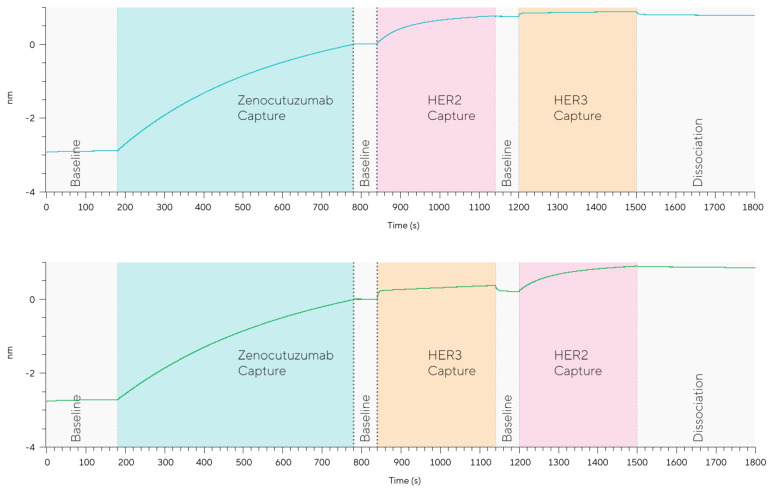
Sequential binding of HER2–HER3 (**top**) and HER3–HER2 (**bottom**) shows zenocutuzumab is capable of binding both antigens sequentially using the dual binding assay format. Sensorgrams normalized to zenocutuzumab capture response.

The dual binding assay was repeated as above with sequential binding of HER2-HER3 or HER3–HER2. As shown in Figure 12 (top and bottom respectively), zenocutuzumab can bind HER2-HER3 and HER3–HER2 sequentially with a similar final binding response (normalized to zenocutuzumab capture response).

**Figure 13:**
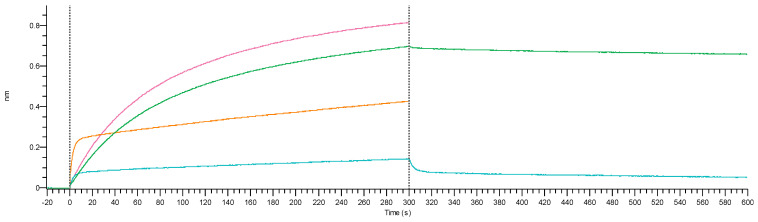
Analysis of the final binding response for HER2–HER3 and HER3–HER2 sequential assessment reveals markedly different ratios of antigen binding, which is dependent upon which antigen binds first. Dotted line indicates the start of the dissociation step.

Although the final binding response observed for sequential binding was similar, further assessment of the data using single reference subtraction shows that the constituent parts of the final binding response are markedly different (Figure 13 and Table 4).

Sequential HER2–HER3 binding shows a very similar binding response of HER2 when compared to individual binding (91.3%) but a binding response of only 32.5% for HER3 when compared to individual binding (Table 4). Analysis of the reverse antigen sequence HER3–HER2 exhibits a similar effect with HER3 exhibiting a binding response of 96.5% compared to individual binding but HER2 shows a higher binding response of 78.1% when assessed after HER3. The difference in percentage binding response of the secondary antigen may, in part, be due to the higher affinity of zenocutuzumab for HER2 but may also be due to allosteric effects between the HER2 and HER3 variable domains. Allostery between domains would not be unexpected as inter-domain cooperativity has been shown to affect antigen binding [18,19] and allostery between the variable and constant region upon antigen binding is a well-known phenomenon and may play a role in the observed enhanced ADCC for zenocutuzumab [14].

Gassner et al. and Meschendoerfer et al. showed that both the bridging and dual binding assay formats can be used to determine the individual binding components of a purified bispecific and loss of binding can be attributed to loss of either antigen paratope [16,17].

Assessment of correct bispecific formation during initial protein engineering and optimization is essential to ensure the production of a correctly folded bispecific antibody that can bind both antigens. Therefore, the dual binding assay format was adapted to include a loss of function in order to show that Octet^®^ BLI can determine the amount of bispecific antibody in a solution that contains both antigen binding paratopes.

Heterodimeric IgG (HC + LC) Her2-hOKT3 BsAb-L (bAb0526) is a bispecific antibody that contains the HER2 binding paratope of trastuzumab and the CD3 binding paratope of OKT3(Absolute Antibody). A titration of bAb0526 into a decreasing concentration of zenocutuzumab was performed in order to simulate different ratios of bispecific antibody that may be encountered during protein production (Table 5). As both zenocutuzumab and bAb0526 contain HER2 binding paratopes it would be expected that the binding response of HER2 should remain similar whilst the binding response of HER3 should decrease due to the replacement of the HER3 binding paratope in zenocutuzumab with the CD3 binding paratope in bAb0526.

**Figure 14:**
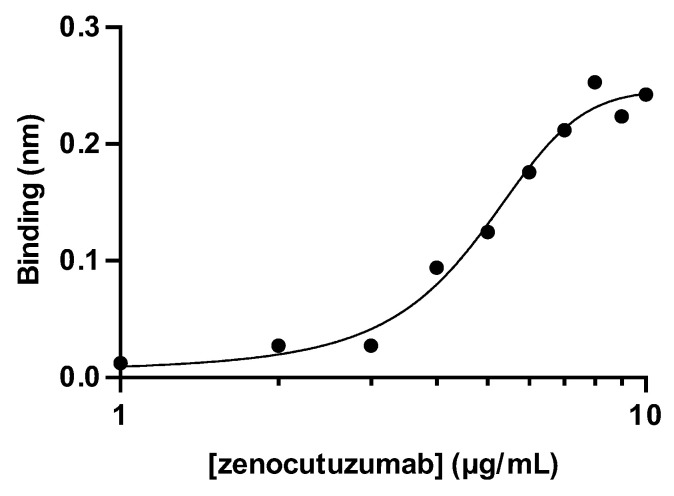
Dose–response curve for HER3 binding with a decreasing zenocutuzumab concentration. HER2 binding remained consistent due to an increasing titration of bAb0526.

As shown in Figure 14, the dual binding assay format could detect the decreasing titration of zenocutuzumab and the corresponding decrease in the HER3 binding response due to the loss of the HER3 paratope. The HER2 binding response remained stable throughout the assay with an average binding response of 0.856 nm (%CV 7.1%). Therefore, the dual assay format is suitable for assessment of bispecific antibodies during protein engineering and optimization as the assay format has the sensitivity to detect a change in the concentration of antibody present. As shown in Figure 14, a 50% decrease in zenocutuzumab corresponds to a 50% lower binding response than 100% zenocutuzumab (0.1247 nm vs. 0.2425 nm, respectively), which corresponds to the linear dose–response phase of the logarithmic plot of the binding response of zenocutuzumab.

## 5. Detecting Empty and Full Adeno-Associated Virus (AAV) Using the Octet^®^ BLI

Adeno-Associated virus (AAV) is composed of an icosahedral protein capsid of ~26 nm in diameter and a single-stranded DNA genome of ~4.7 kb that can either be the plus (sense) or minus (anti-sense) strand. The AAV genome consists mainly of two viral genes: replication (rep) and capsid (cap), flanked by inverted terminal repeats (ITRs). The ITRs have a palindromic nucleotide sequence and create characteristic T-shaped hairpin structures, providing essential structural elements for viral genome replication and packaging. The open reading frame (ORF) of rep encodes several nonstructural proteins that are required for gene regulation, replication, transcription, and encapsidation. The ORF of cap encodes three structural proteins including virion protein 1 (VP1), VP2, and VP3. Distinct tissue tropism of different AAV serotypes results from variations in the processing of this cap ORF, leading to variant immune and transduction profiles. Different serotypes therefore have tropism for specific organs and tissues of the body and can be selectively deployed in gene therapy to target different organs [20,21,22].

In gene therapy, gene replacement is considered as a key strategy; it aims to deliver a gene product to compensate for loss of function mutations in various disease states [23] and AAV vectors are the leading platform for gene delivery for the treatment of a variety of human diseases. During development and production, AAV in-process samples often yield mixed populations of particles that include full capsids, partially full capsids and empty capsids, amongst others. This heterogeneity during production necessitates efficient product characterization to establish the empty-to-full capsid ratios. While there are many techniques that have been deployed to detect these ratios, they are often either expensive or time consuming, in addition to many other challenges, hence making it difficult for developers to identify an ideal cost-effective technique.

The Octet^®^ BLI AAVX biosensor was developed to enable rapid, real-time and high-throughput measurement of AAV capsid titer in samples across the AAV workflow, enabling quick process optimization, quality checks and increased productivity. The biosensors have broad serotype-binding specificity, allowing for the quantitation of 10 different serotypes. BLI principles of detection have been utilized to extend the use of these biosensors to empty versus full capsid detection.

### 5.1. Principles for Empty vs. Full Detection of AAV Capsids Using the Octet^®^ AAVX Biosensor

**Figure 15:**
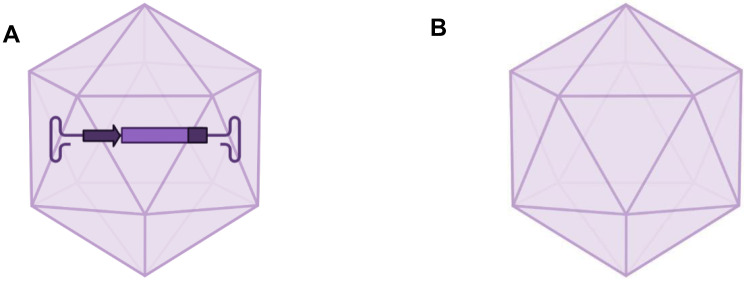
Schematic of full AAV capsid with genomic insert (**A**) and empty capsid (**B**) with no insert. At an equivalent capsid titer, the two exhibit different binding saturation levels on Octet^®^ AAVX biosensors that can be used for the determination of empty vs. full populations in an AAV bioprocess sample.

As shown in Figure 1, the principles of BLI technology are such that binding on the tip of the biosensor results in a molecular layer which increases in thickness as more analyte molecules bind to the surface. The spectral pattern changes as a function of the optical impedance of the molecular layer is monitored at the detector. While the AAVX biosensor utilizes an anti-AAV antibody to bind to capsid proteins, the principles of detection of empty capsids relative to full capsids on BLI capitalizes on the fact that, although both bind to the antibody via the proteins on the surface of the capsids, the genomic insert in the full capsid imparts a density difference between the two (Figure 15). This leads to a difference in the binding levels at saturation when bound to the antibody on the biosensor surface.

**Figure 16:**
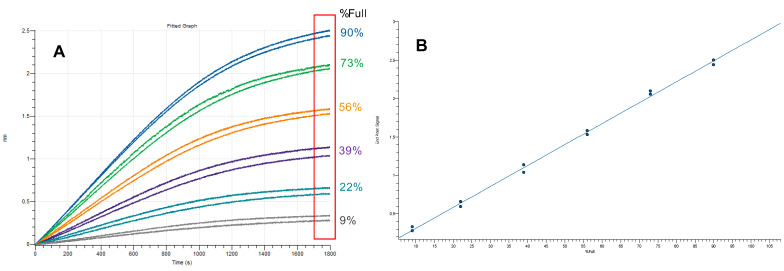
Binding response of AAV8 in sample diluent buffer (**A**) and at different % full ratios (90, 73, 56, 39, 22, 9) with the resulting standard curve (**B**). Samples were obtained as empty and full reference standards from Progen Catalogue numbers 66V080 and 66V081, respectively. Both full and empty capsids were first tittered at 2.5 × 10^11^ cp/mL and then mixed to the targeted % full samples.

As a result, at a nominally equivalent capsid titer, 100% full genomic capsids will exhibit a binding response level on the biosensor surface that is higher than an equivalent amount of 100% empty capsids at the biosensor binding saturation. As a result, the difference in BLI signal is solely due to density differences between equal numbers of AAV particles, with samples of higher percentage full generating higher signals (Figure 16). A standard curve can thus be established by generating binding response data from a mixture of high and low percentage full reference materials to determine the empty-to-full capsid ratio of unknown samples in a typical Octet^®^ BLI dip-and-read assay. This method is rapid, easy to perform, and accommodates a wide variety of matrices, including untreated crude cell lysate, providing an attractive solution to both upstream and downstream development efforts in AAV manufacturing. By analyzing intact viral capsids, the assay eliminates the need to release the packaged DNA, which frequently underestimates viral genome titer due to DNA loss during sample treatment. Furthermore, the saturation-based analysis is more tolerant to minor differences in sample titer, thereby eliminating compounded error, a limitation intrinsic to other methods that measure viral genome and capsid titers separately; the main requirement, however, is that the capsid titer be (≥2 × 10^11^ vp/mL). Since the method assumes binding saturation on the biosensor surface, a lower titer may be usable with a longer assay time (not evaluated).

### 5.2. Matrix and Serotype Compatibility of the Assay

**Figure 17:**
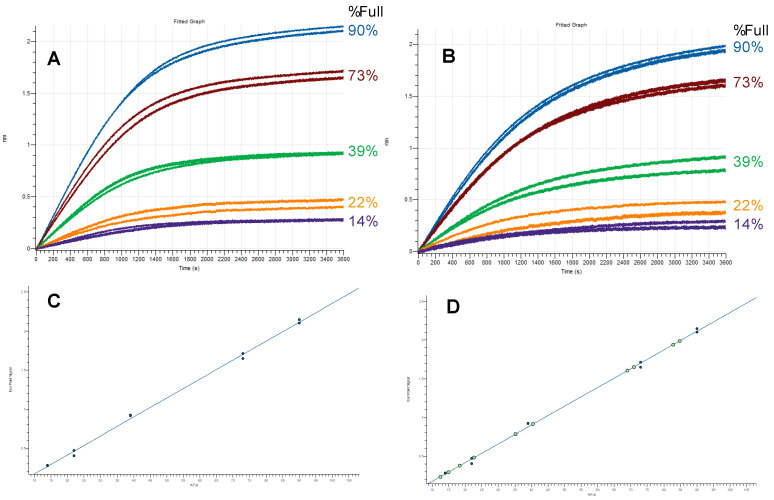
Assay matrix compatibility using AAV8 samples spiked in Octet^®^ sample diluent buffer (**A**) and in crude cell lysate (**B**) and the respective standard curves of binding as a function of % full capsids (**C**,**D**).

The Octet^®^ AAVX biosensor was developed with broad specificity and is designed for use with multiple AAV serotypes including AAV1, AAV2, AAV3, AAV4, AAV5, AAV6, AAV7, AAV8, AAV9 and AAVrh10. However, when used in genomic insert detection for comparison between empty and full capsids, the biosensor was optimized for AAV2, AAV5 and AAV8 serotypes. Figure 17 shows binding curves of AAV8 serotype in different matrices with the required dilution schemes shown in Table 6. Other serotypes may require user optimization keeping in mind assay conditions requirements as stipulated in the Octet^®^ AAVX biosensor technical note [24]. Since this assay takes advantage of the density differences between full and empty capsids, the genomic insert’s size and the normalized capsid titer are the key determinants to achieving a robust assay.

## 6. Conclusions

Octet^®^ BLI technology is widely accepted as a key platform for quantitation and kinetic characterization of antibodies and other large molecular weight biologics. In these applications, the assay workflow revolves around a simple dip-and-read process. The biosensors are either dipped into the capture ligand followed by a dip into the analyte or, in the case of off-the-shelf ligand-coated biosensors, dipped directly into the analyte. While this process works well for most analytes, it is not sensitive enough to detect lowly abundant molecules. In addition, other biological modalities, including bispecific and multispecific antibodies, liquid nanoparticles (LNPs), and viral particles used as drug delivery agents, may have been considered out of reach for BLI-based technologies. However, this article demonstrates that, with proper assay design and the appropriate assay optimization, Octet^®^ BLI can be extended for use in these less traditional applications.

It is important to note that low analyte detection has previously been established on BLI for the detection of residual host cell proteins (HCP) and that it is a well-established application on SPR platforms due to SPR technology’s higher sensitivity; however, the routine use of BLI for analytes such as cytokines and other biomarkers is not well established [25]. This article demonstrates that with optimization these assays can be routinely run on BLI. The article outlines the development and optimization of a multi-step Octet^®^ BLI assay that utilizes an enzyme signal amplification step for the detection of cytokines (TNFα and IL-6). The introduction of a combined sample and HRP-conjugated antibody step alongside AEC amplification resulted in increased signal magnitude and improved sensitivity over traditional single-step assays. In these experiments AEC is established as a preferred detection reagent over the more commonly used metal-enhancing DAB as it demonstrates higher sensitivity detection. The optimization of various parameters, including capture and detection antibody concentration, conjugation ratios and assay shake speeds, contributed to reproducible quantitation of low cytokine concentrations. While it is noted that there are other label-free analytical techniques with the sensitivity to detect sub-ng/mL concentrations of low abundance analytes [26], this sensitivity range is typically considered more challenging for a mass-based technology such as BLI principally due to the mechanisms of detection. It should also be noted that although traditional ELISA-based kits may offer improved sensitivity over label-free techniques, they also require extensive hands-on time and time-to-result of approximately 90 min and 270 min, respectively [27]. This is in stark contrast to the single plate ‘walk-away’ method described here that has minimal hands-on time and a time-to-result of less than 30 min.

The enhanced sensitivity and reproducibility of the BLI multi-step assay offers BLI users a valuable tool for in vitro research, therapeutic evaluation, and predicting inflammatory responses. The methodology described in this document provides a foundation for further exploration and application to a broader range of molecules beyond cytokines, potentially accelerating advancements in biomedical research and diagnostics.

This document also outlines the use of Octet^®^ BLI for simultaneous analysis of multiple molecular interactions, as exemplified by quantitation of mixed populations of bispecific antibodies. While the bispecific antibody quantitation approach may require a further deep dive, the data generated and the suggested method indicate that it may complement the use of more sophisticated techniques for bispecific antibody detection and quantitation. Finally, we have taken advantage of BLI density-based principles for quantitation of AAV empty-to-full ratios in mixed populations of capsids. While optimization may be needed for certain serotypes and matrix conditions, the proposed method avoids the lengthy and often inefficient capsid lysis step required to expose the ssDNA for binding and subsequent detection, by comparative methods. The only critical requirements are that (1) the genomic insert is large enough to yield sufficient signal differences between empty and full genomic capsids, (2) the capsid titer of unknown samples is adjusted to match the standards, and (3) standard or reference samples against which empty vs. full capsid ratios are determined are well characterized in terms of both titer and percentage full to ensure accuracy. The method described in this article is a proof-of-concept method that can be further evaluated as an orthogonal technique to more established and industry-accepted methods such as analytical ultracentrifugation (AUC) and cryo-electron microscopy (cryo-EM) and could extend BLI usage into the cell and gene therapy space.

## Figures and Tables

**Table 1:** AEC reproducibility and curve fit parameters. Three replicates were run on the same sample plate.

Known Concentration (pg/mL)	Calculated Concentration CV (%)	Recovery (%)	Residuals	R^2^
50,000	1.4	100.0	8.55	1.000
16,667	2.6	100.1	11.37	1.000
5556	2.7	98.4	7.39	0.998
1852	3	106.6	2.30	0.992
617	2.4	110.7	1.80	1.000
206	4.2	111.6	9.67	1.000
69	3.5	99.4	2.53	0.994

**Table 2:** Metal-enhancing DAB reproducibility and curve fit parameters. Three replicates were run on the same sample plate.

Known Concentration (pg/mL)	Calculated Concentration CV (%)	Recovery (%)	Residuals	R^2^
50,000	1.3	100.0	0.94	0.997
16,667	5.1	100.3	3.76	0.999
5556	4.4	95.4	4.63	0.998
1852	1.9	113.9	13.93	0.996
617	2.8	146.7	46.65	0.988
206	9.2	142.9	43.10	0.968
69	49	99.4	43.45	0.921

**Table 3:** Binding response taken as an average over 290–295 s.

Individual Binding Response (nm)		Sequential Binding Response (nm)			
		HER2 then HER3		HER3 then HER2	
HER2	HER3	HER2	HER3	HER3	HER2
0.8884	0.4398	0.8109	0.1431	0.4244	0.6935

**Table 4:** The final antigen ratio is dependent on the sequence of antigen binding.

Individual Binding Response (%)		Sequential Binding Response (%)			
		HER2 then HER3		HER3 then HER2	
HER2	HER3	HER2	HER3	HER3	HER2
100	100	91.3	32.5	96.5	78.1

**Table 5:** An initial starting concentration of 10 µg/mL of zenocutuzumab was assessed for binding to HER2 and HER3. bAb0526 was titrated into the capture solution with a corresponding decrease in zenocutuzumab.

Concentration of Zenocutuzumab (µg/mL)	Concentration of bAb0526 (µg/mL)
10	0
9	1
8	2
7	3
6	4
5	5
4	6
3	7
2	8
1	9
0	10

**Table 6:** Method compatibility with some common AAV sample matrices. The observed sample matrix compatibility of the Octet^®^ biosensor saturation method for empty vs. full AAV capsid detection makes this method highly suitable for a wide range of AAV purification workflows. * Reagent from Thermo Fisher Scientific.

Matrix Category	Matrix Type	AAV Serotype Tested	Recommended Dilution Factor
Buffer	Octet^®^ Sample Diluent	AAV2/5/8	Neat
Culture media	Chemically defined, protein free medium (Viral Production Media *, FreeStyle 293 Expression Media *)	AAV5/8	Neat
	DMEM + 10% Fetal Bovine Serum	AAV5/8	5-fold in Octet^®^ Sample Diluent
Cell lysis solutions	1× Lysis Buffer with 1% Tween-20 in protein-free culture media	AAV5	Neat
	0.5 M NaCl in protein-free culture media	AAV5/8	Neat
Cell lysate	2 mg/mL HEK293 cell lysate with 1× Lysis Buffer and 0.5 M NaCl	AAV5/8	Neat

## Data Availability

Data other than that shared in the document is unavailable as the research shared herein originates from Sartorius’s internal applications development work.
